# Implementation of couples’ voluntary HIV counseling and testing services in Durban, South Africa

**DOI:** 10.1186/s12889-015-1959-z

**Published:** 2015-07-02

**Authors:** William Kilembe, Kristin M. Wall, Mammekwa Mokgoro, Annie Mwaanga, Elisabeth Dissen, Miriam Kamusoko, Hilda Phiri, Jean Sakulanda, Jonathan Davitte, Tarylee Reddy, Mark Brockman, Thumbi Ndung’u, Susan Allen

**Affiliations:** Rwanda Zambia HIV Research Group, Department of Pathology & Laboratory Medicine, School of Medicine, Emory University, Atlanta, GA USA; Hubert Department of Global Health, Rollins School of Public Health, Emory University, Atlanta, GA USA; Department of Epidemiology, School of Public Health, Emory University, 1518 Clifton Road NE, 4011, Atlanta, GA 30322 USA; HIV Pathogenesis Programme, Doris Duke Medical Research Institute, University of Kwa Zulu Natal, Durban, South Africa; Medical Research Council, Biostatistics unit, Durban, South Africa; Faculty of Health Sciences and Faculty of Science, Department of Molecular Biology and Biochemistry, Simon Fraser University, British Columbia, Canada; KwaZulu-Natal Research Institute for Tuberculosis and HIV (K-RITH), University of KwaZulu-Natal, Durban, South Africa

**Keywords:** Heterosexual couples, Couples’ HIV voluntary counseling and testing, South Africa, HIV prevention, Implementation science

## Abstract

**Background:**

Couples’ voluntary HIV counseling and testing (CVCT) is an evidence-based intervention that significantly reduces HIV incidence in couples. Despite the high prevalence of HIV and HIV couple serodiscordance in South Africa, there are few CVCT services.

**Methods:**

From February-June 2013, The Rwanda Zambia HIV Research Group provided support, training, and technical assistance for local counselors and promoters to pilot CVCT services in five hospital-based clinics in Durban, South Africa. Client-level data (age, gender, years cohabiting, pregnancy status, previous testing, antiretroviral treatment (ART) status, neighborhood, and test site) collected as a component of routine CVCT service operation is presented stratified by couple serostatus.

**Results:**

Twenty counselors and 28 promoters completed training. Of 907 couples (1,814 individuals) that underwent CVCT, prevalence of HIV was 41.8 % and prevalence of HIV serodiscordance was 29.5 % (19.3 % M-F+, 10.3 % M + F-). Most participants were 25–34 years of age, and this group had the highest prevalence. Previous individual HIV testing was low (50 % for men, 63 % for women). Only 4 % of couples reported previous CVCT. Most (75 %) HIV+ partners were not on ART, and HIV+ individuals in discordant couples were more likely to be on ART than those in concordant positive couples. Pregnancy among HIV+ women was not associated with previous HIV testing or ART use.

**Conclusions:**

Implementation of standard CVCT services was found to be feasible in Durban. The burden of HIV and couple serodiscordance in Durban was extremely high. CVCT would greatly benefit couples in Durban as an HIV prevention strategy.

## Background

Almost two-thirds of incident HIV infections in sub-Saharan Africa occur among stable concordant HIV negative or HIV discordant (one partner testing HIV seropositive and one partner testing HIV seronegative) couples [7]. In an analysis of Demographic and Health Surveys data from 24 sub-Saharan African countries, HIV incidence among concordant HIV negative couples contributes to roughly 30 % of all new infections while incidence among discordant couples contributes another 30 % [7]. About half of all HIV-infected persons in the region are in discordant relationships [13], and the majority of transmissions between serodiscordant couples originate with the HIV-positive index, not concurrent, partners [6].

Couples’ voluntary HIV counseling and testing (CVCT) is associated with a two-thirds reduction in incidence in discordant couples, reduced incidence of new infections in concordant HIV negative couples, and reductions in sexually transmitted infection (STI), mother-to-child transmission (MTCT), and unplanned pregnancies for all couples [3–5, 10, 15, 17, 18, 20, 24].

In South Africa, most HIV is transmitted heterosexually. Nation-wide HIV prevalence among persons aged 15–49 is estimated at 23.3 % for women and 13.3 % for men and varies considerably by region. KwaZulu-Natal has the highest HIV prevalence among those aged 15–49, estimated at 27.6 % by the 2012 HSRC survey [33]. Additionally, the Partners in Prevention HSV-2/HIV-1 Transmission study group reported a very high prevalence of serodiscordance in South African couples of 27.4 %, much higher than other countries in the region [28]. A recent study in Soweto (in the Johannesburg metropolitan area) comprised of 11,163 sexually-active HIV-infected men and women in an urban primary care program indicated that 40 % of participants had a known positive partner, while 40 % of participants had a partner of unknown HIV status and 20 % had an HIV-negative partner. Participants in this study had been in their relationships for a substantial period of time (median >3 years), and knowledge of partner HIV status did not vary by the duration of relationships [38].

These high rates of HIV prevalence and HIV serodiscordance reinforce WHO recommendations for couples-based HIV testing, which is not yet widely available in South Africa. The Rwanda Zambia HIV Research Group (RZHRG) has been providing CVCT services in Rwanda and Zambia for over 20 years and has provided support and training for CVCT under a CDC Centers of Excellence grant to over 20 countries, including South Africa [1, 32]. RZHRG has a successful history of recruiting couples for CVCT and building capacity within local communities to provide the service [2, 27, 39].

The implementation of CVCT was recently piloted in five health facilities in Durban, South Africa. During this pilot, RZHRG provided on-site support, training, and technical assistance to health facility staff; trained local clinic promotions staff to mobilize their communities to attend CVCT; assisted with implementation of the service; and helped monitor and evaluate patient-level data including testing outcomes, antiretroviral treatment (ART) use, and pregnancy status. Here, we present the implementation methods as well as CVCT client-level outcomes among couples receiving CVCT in Durban during this pilot. The “pilot” refers to all activities related to CVCT promotions and service provision.

## Methods

### Didactic and practical training for local clinic counselors in the CVCT intervention

The Canadian Sub-Saharan Africa HIV/AIDS Network sponsored the study, which was a collaboration between RZHRG and the HIV Pathogenesis Programme (HPP) in the Doris Duke Medical Research Institute (DDMRI) at the University of KwaZulu-Natal’s Nelson R. Mandela School of Medicine Campus. Five hospital-based clinics in Durban were selected for the pilot, and selection was based on large catchment population and proximity to HPP. All five clinics are located in the biggest township in Kwa Zulu Natal Province called Umlazi. Umlazi is 4,481 hectares in size and has a population estimated at 550,000 m to over 1 million with approximately 30 % of people living in informal housing [21]. It is divided into 26 community sections using alphabets from A to Z with additions of AA, BB, CC. HPP and Gateway clinic are both located on the grounds of Prince Mshiyeni Memorial Hospital (PMMH). Whereas HPP is a research center that follows up participants recruited in a variety of research studies, Gateway provides outpatient care services to people in the catchment of PMMH and mainly the V-section of Umlazi. D, H and V clinics are all small clinics within a 20 km radius of HPP and all provide outpatient care services to the respective communities in D, H and V sections of Umlazi. Except for HPP, all the other clinics are managed by nurses and counselors and have a visiting doctor who goes to the clinic to provide HIV care and treatment. HPP is has research staff that include a physician, nurses, counselors and administrative staff. All clinics are of average size (8–10 rooms used for services and waiting areas) with the exception of V-clinic which is smaller (about 4 rooms). A more detailed description of the clinics is provided in Table 1.

**Table 1:** Description of clinics selected for CVCT implementation, Durban, South Africa, 2013

	HPP PMMH Parkhome	Gateway clinic	D clinic	H clinic	V clinic
Service area	The HPP PMMH Parkhome is based on the grounds of PMMH Hospital. Participants from HPP research cohorts attend their follow up visits here. This clinic mainly services the V section community.	This clinic is based on the grounds of PMMH Hospital and services the V section community.	This clinic mainly services the D section community.	This clinic mainly services the H section community.	This clinic mainly services the V section community.
Primary service provided	Clinic services are mainly focused on follow-up of participants recruited into the different HPP studies.	Treatment of minor ailments, ANC, ART, VCT, TB screening, immunizations.	Treatment of minor ailments, ANC, ART, VCT, TB screening, immunizations.	Treatment of minor ailments. Immunizations.	Treatment of minor ailments, ANC, ART, VCT, TB screening, immunizations.
Staffing	Permanent staff consists of ne nurse, one counsellor, one doctor, and one site administrator.	Staff is a compliment of nurses and counsellors. A doctor visits on Mondays for ART provision.	Staff is a compliment of nurses and counsellors.	Staff is a compliment of nurses and counsellors.	Staff is a compliment of nurses and counsellors. A doctor visits on Mondays for ART provision.

In February 2013, three RZHRG counselor trainers, each with over ten years of experience in providing CVCT and CVCT trainings, conducted didactic and practical CVCT training sessions for groups of local clinic counselors. These five day trainings were developed with US CDC [8] and adapted for site-specific use through a CDC Centers of Excellence grant. Whenever training is provided in a new setting, the CDC materials are translated and adaptation as needed to the local circumstances. Didactic trainings include modules on understanding HIV discordance; couple counseling skills; the CVCT intervention; data collection; laboratory guidelines; providing concordant negative results, concordant positive results, and discordant results; and providing counseling on support and prevention services. A portion of the last day of the training was used to discuss the trainees’ expectations and perceptions of the training curriculum and to address any concerns trainees may have regarding working in the field. All counselors were given pre- and post-tests assessments prior to and immediately following the didactic training, with a score of 75 % at posttest required in order to proceed with practicum training. RZHRG counselor trainers supervised CVCT counselor trainee practicums, which were conducted the week after the didactic training. Practicums required each counselor to observe at least two counseling sessions led by the RZHRG trainers and execute at least two supervised counseling sessions for each possible couple-level HIV serostatus result (concordant negative, concordant positive, discordant).

### Didactic and practical training for local community health volunteers to promote CVCT

Simultaneous with the counselor trainings, two RZHRG promotions trainers with extensive experience promoting CVCT among communities in Zambia conducted a three day didactic training for CVCT promotions for local volunteers from the community. These volunteers were identified and screened based on previous experience working in health promotional activities. Didactic trainings include modules on an introduction to CVCT and the promotion of CVCT with delivery of invitations, the role of promoters, promotional strategies and promoter webs of influence, and CVCT promotional skills including public speaking and communication skills. Promoters were also trained in data collection during invitation delivery. A portion of the last day of the training was used to discuss the trainees’ perceptions of the training curriculum and to address any concerns they may have in working in the field. All community health volunteers were given pre- and post-training assessments. Scores of 65 % were required to be certified as competent. This threshold score for qualification for promoters is less stringent than that required for counselors because the promoter’s role is to provide basic information about the importance of HIV testing that encourages couples to access CVCT. Generally they have a lower level of education than counselors or nurses. The counselor’s role is more technical and the accepted score for certification is higher to ensure that only counselors that are likely to provide good quality service are allowed to practice CVCT. RZHRG promotions trainers then observed field practicums as trainee promoters began the process of inviting couples to attend pilot weekend CVCT services. Each promoter invite card was coded to allow for tracking and performance-based reimbursement. Promoters used one-to-one, door-to-door, group presentation, and clinic health talks to advocate for CVCT and encourage couples to use the service. They gave out invitations mostly in the communities they lived which are the catchment areas of the five clinics; however they were not restricted to these areas and were free to invite from other areas of Umlazi.

### Data training on quality control and cleaning of databases

Client-level health indicators were collected for tested couples as a component of routine CVCT service operation including: age, gender, years cohabiting, pregnancy status, previous HIV testing, previous CVCT, ART use, neighborhood, and HIV serostatus of both partners. The HIV testing algorithm was based on the South African algorithm using two rapid HIV test kits (Alere Determine HIV-1/2 and Uni-Gold Recombigen HIV-1/2) run in parallel for each individual. The RZHRG team has developed data-collection tools to systematically and accurately collect these indicators. These tools have been used extensively by government clinic staff in Zambia and were easily adapted for use in this pilot. Collection of this data using electronic databases already established in RZHRG’s Zambia and Rwanda programs were used to record client indicators throughout the CVCT pilot. Counselors were trained on collecting data and quality control of data in the field for all data collection instruments including CVCT monitoring and evaluation log books, aggregate data sheets, and promoter information log books. Data entry into Microsoft Access databases, data quality control, and data cleaning was performed by a data manager in the Durban office after training by the RZHRG data manager.

### Pilot CVCT services in five hospital-based clinics

Over twelve weekends (Saturday and Sunday) from March to June 2013, CVCT services were offered in the five pilot clinics from 8 am to 5 pm. Couples presented at the clinic with an invitation from a promoter and either attended a group session i.e. if more than one couple was available or went straight into pre-test counseling. They were then tested for HIV and given results and posttest counseled together. RZHRG trainers were available to support the clinics during this period in all aspects of the program (i.e., counseling, promotion and data management). The timeline of implementation events is shown in Table 2.

**Table 2:** Timeline of CVCT implementation events, Durban, South Africa, 2013

Week 1	Didactic training of 1st cohort of counselors (13 counselors from each of the 5 clinics)
	Prepare clinic supplies and data collection materials
	Training and practicum for promoters
Week 2	Didactic training of 2nd cohort of counselors (8 counselors from each of the 5 clinics)
	Practical training of 1st cohort of counselors
	Promoters finish practical and do promotions under supervision
Week 3	Clinics open with 1st cohort of counselors
	Practical training of 2nd cohort of counselors
	Promotions
	Data training on quality control and cleaning of databases, observe data collection in clinics
Weeks 4-20	Clinics open with all trained counselors
	Counselors facilitate clinic talks in antenatal care and under five clinics
	Promotions
	Data entry, quality control, and cleaning

### Data analysis

Client-level indicators (age, age-disparity between partners, cohabitation status and duration, pregnancy status, previous HIV testing, previous CVCT, ART status, neighborhood, and test site) were analyzed with descriptive statistics (means and standard deviations (SD) for continuous variables; counts and frequencies for categorical variables). All variables were stratified by couple HIV serostatus results (concordant positive, discordant man positive, discordant woman positive, and concordant negative) and differences in the distribution of client-level indicators by HIV serostatus categories were evaluated with Chi-square (or Fisher’s exact) tests or ANOVA, as appropriate. We also explored previous testing and ART use among women by pregnancy status. Analyses were conducted with SAS v9.4 (Cary, NC) and all p-values are two tailed.

### Ethics statement

The study was approved by the Biomedical Research Ethics Committee of the University of KwaZulu-Natal. Written informed consent was not required from couples obtaining CVCT services. Couples provided oral consent to be tested as a couple after the group counselling and individual pre-test counseling sessions.

## Results

### CVCT recruitment and counselors

Trained recruiters recruited couples throughout the whole of Umlazi and referred them to one of the five clinics. Recruiters were not limited to a catchment area, though focused on recruiting from homes, markets, and taxi ranks around the clinics.

Twenty-one individuals (18 females and 3 males) that completed the training, provided CVCT to the recruited couples in the five clinics. The majority were counselors and there were 2 nurses and 1 registered midwife. All had at least matriculation level of education (i.e., completion of the final year of high school and all qualifications necessary for university entrance) and their median years of experience was 6 years (range from 1–10 years).

### Client-level outcomes (Table 3)

**Table 3:** Demographic characteristics by testing outcomes of CVCT participants, Durban, South Africa, 2013

	Total couples (*N* = 907)		Couple serostatus								Chi-square *p*-value (2-tailed)^a^
			M + F+ (*N* = 245)		M + F- (*N* = 93)		M-F+ (*N* = 175)		M-F-(*N* = 394)		
	N	col %	N	col %	N	col %	N	col %	N	col %	
Man age (mean, SD)	30.8	8.4	33.2	7.3	32.4	9.0	30.5	7.4	29.0	9.0	<0.0001
Man age											<0.001
<25 years	181	20 %	17	7 %	13	14 %	30	17 %	131	33 %	
25-34 years	481	53 %	127	52 %	51	55 %	115	66 %	188	48 %	
≥35 years	245	27 %	101	41 %	29	31 %	40	23 %	75	19 %	
Woman age (mean, SD)	28.4	7.9	30.2	6.72	30.4	8.9	28.9	6.7	26.6	8.51	<0.0001
Woman age											<0.001
<25 years	308	34 %	46	19 %	25	27 %	39	22 %	198	50 %	
25–34 years	438	48 %	144	59 %	45	48 %	106	61 %	143	36 %	
≥35 years	161	18 %	55	22 %	32	34 %	30	17 %	53	13 %	
Age disparity (mean, SD)	3.4	3.0	4.4	3.9	3.2	2.3	3.5	3.0	3.4	3.0	<0.001
Age disparity											0.01
0–2 years	447	49 %	102	42 %	47	51 %	93	53 %	205	52 %	
3–4 years	203	22 %	50	20 %	26	28 %	38	22 %	89	23 %	
5–6 years	120	13 %	37	15 %	12	13 %	22	13 %	49	12 %	
>6 years	137	15 %	56	23 %	8	9 %	22	13 %	51	13 %	
Years cohabiting (mean, SD)	1.5	3.7	1.8	3.6	2.3	5.1	1.4	2.4	1.2	3.8	<0.001
Years cohabiting											<0.001
Not cohabiting	602	67 %	144	60 %	45	48 %	105	60 %	308	78 %	
≤2 years	136	15 %	42	17 %	20	22 %	35	20 %	39	10 %	
>2 years	164	18 %	55	23 %	28	30 %	35	20 %	46	12 %	
Couple pregnant											0.26
Yes	95	11 %	21	9 %	14	15 %	22	13 %	38	10 %	
No	809	89 %	222	91 %	79	85 %	153	87 %	355	90 %	
Man previously tested											0.41
Yes	449	50 %	116	48 %	52	56 %	81	46 %	200	51 %	
No	457	50 %	128	52 %	41	44 %	94	54 %	194	49 %	
Woman previously tested											0.42
Yes	570	63 %	159	65 %	57	61 %	101	58 %	253	64 %	
No	337	37 %	86	35 %	36	39 %	74	42 %	141	36 %	
Couple previously tested together											0.52
Yes	36	4 %	8	3 %	3	3 %	5	3 %	20	5 %	
No	870	96 %	237	97 %	89	97 %	170	97 %	374	95 %	
ART use among HIV positive couples									*n/a*		0.95^
Man on ART	27	5 %	7	3 %	20	22 %	*n/a*				
Woman on ART	75	15 %	40	16 %	*n/a*		35	20 %			
Both partners on ART	26	5 %	26	11 %			*n/a*				
Neither on ART	382	75 %	170	70 %	72	78 %	140	80 %			
Neighborhood recruited											0.16
D Clinic	238	27 %	67	28 %	21	23 %	42	25 %	108	28 %	
H Clinic	188	21 %	50	21 %	18	19 %	40	24 %	80	21 %	
HPP	121	14 %	28	12 %	11	12 %	25	15 %	57	15 %	
Gateway Clinic	233	26 %	56	23 %	32	34 %	51	30 %	94	24 %	
V Clinic	110	12 %	39	16 %	11	12 %	10	6 %	50	13 %	
CVCT test site											<0.001
D Clinic	181	20 %	54	22 %	12	13 %	29	17 %	86	22 %	
H Clinic	134	15 %	41	17 %	7	8 %	25	14 %	61	15 %	
HPP	88	10 %	30	12 %	5	5 %	18	10 %	35	9 %	
Gateway Clinic	389	43 %	79	32 %	58	62 %	94	54 %	158	40 %	
V Clinic	115	13 %	41	17 %	11	12 %	9	5 %	54	14 %	

^a^differences between serostatus groups

^*p*-value for HIV positive individuals on ARV versus not

*SD*: standard deviation; *ART*: antiretroviral treatment; *HPP*: HIV Pathogenesis Program Research Clinic

Of the 907 couples (1,814 individuals) that underwent CVCT, the prevalence of HIV was 41.8 % and the prevalence of HIV serodiscordance was 29.5 % (19.3 % M-F+, 10.3 % M + F-). 245 (27 %) of couples were concordant positive and 56.6 % of all couples had at least one partner who was HIV positive. Of all HIV positive persons, 35.4 % had HIV negative partners. The pilot identified at least 313 new HIV positive individuals, and possibly more if previous tests were HIV negative (we did not ask clients their HIV results from previous testing).

Men were on average 30.1 (SD = 8.4) years of age, women were on average 28.4 (SD = 7.9) years of age, and most participants were aged between 25–34 years. Concordant positive couples were significantly older on average than concordant negative couples; discordant M + F- couples were also significantly older on average than concordant negative couples (p < 0.001). The mean age difference between partners was 3.7 (SD = 3.2) years, and age disparity was significantly higher in concordant negative couples (mean 4.4 (SD = 3.9) versus other couples).

Most (67 %) couples were not cohabiting; those that were had been cohabiting for 4.5 (SD = 5.2) years on average. Concordant negative couples were the least likely to be cohabiting while M + F- couples had been cohabiting the longest. Pregnancy status did not differ by serostatus, and 11 % of all couples were pregnant at the time of testing. Previous individual HIV testing was low (50 % for men versus 63 % for women, p < 0.001). Only 4 % of couples reported previous CVCT with no difference by couple serostatus.

Most (80 %) HIV positive individuals were not on ART, and ART use for individuals did not differ significantly by couple serostatus (*p* = 0.95). Most couples were recruited from the D Clinic and Gateway Clinic catchment area neighborhoods. The largest number of couples were tested at the Gateway Clinic site.

### Previous testing and ART use by pregnancy status (Table 4)

**Table 4:** Previous HIV testing and ART use among HIV+ women by pregnancy status

	HIV+ women		HIV+ pregnant women		HIV+ non-pregnant women		*p*-value (2-tailed)
	N =	420	N =	43	N =	377	
	N	col %	N	col %	N	col %	
Previous testing							0.27
Yes	260	62 %	30	70 %	230	61 %	
No	160	38 %	13	30 %	147	39 %	
ART use among women previously tested							
Yes	233	90 %	28	93 %	205	89 %	0.52
No	27	10 %	2	7 %	25	11 %	

*ART*: antiretroviral treatment

Among the *N* = 420 HIV positive women, 10 % were pregnant and 62 % had previously tested for HIV. Among HIV positive women, previous HIV testing was not significantly more common among pregnant versus non-pregnant women (70 % versus 61 %, *p* = 0.27). Of positive women who had been previously tested for HIV, pregnant women were not significantly more likely to be on ART (93 % versus 89 %, *p* = 0.52).

## Discussion

This is the first publication reporting the results of implementing the RZHRG-developed CVCT intervention in an African country outside of Rwanda or Zambia, where the intervention originated and is now a standard of care. Using standardized implementation developed by RZHRG and adapted with the CDC, we were able to quickly and successfully increase capacity for CVCT in Durban. Both counselors and promoters achieved a high level of success in the trainings and in the implementation of CVCT promotion and provision. Each counselor provided services to 7–8 couples/day which was considered to be a feasible workload. The project was well received by the community and health authorities, who saw the importance of encouraging and offering HIV testing to couples and not just individuals.

Though there has been debate about the impact of couples’ HIV testing specifically in South Africa—a country where multiple concurrent partnerships were hypothesized to be a driving factor in the epidemic—a recent population-based data from KwaZulu-Natal suggests that prevalence of multiple or concurrent partnerships among men is currently decreasing [28] and that concurrent partnerships are not an important driver of high HIV incidence in this area [36]. A recent publication of a new randomized controlled trial protocol which aims to increase uptake of CVCT via a group and couple counseling intervention highlights the growing interest in couples-focused HIV prevention. This is the first couples-based intervention with CVCT uptake as its outcome, the results of which may bolster CVCT in South Africa [9]. Couples-focused HIV testing and counseling interventions have also been found acceptable among men who have sex with men in South Africa [33–35]. Additionally, the interest in couples-focused interventions in South Africa is illustrated by the recent results of a pilot study of Project Connect, a HIV/STI prevention intervention developed for US minority couples [11, 12]. Project Connect found that intervention is acceptable among couples in Johannesburg, though not entirely feasible due to stringent eligibility criteria based on previous history of intimate partner violence (IPV) and the number of counseling sessions involved [29]. The investigators of that study recommend that IPV not be an exclusion criterion and that the number of sessions be reduced in order to provide feasible couples-based HIV services. We support both of these recommendations. IPV is a discussion topic in the CVCT trainings as is emphasis on the voluntary nature of the CVCT service.

The promoters were at liberty to give CVCT invitation to individual/couple they encountered in the community regardless of which neighborhood or part of Umlazi the couple came from. The promoter training did not prescribe the areas for invitations to be given but rather put emphasis on strategies for successful invitation. As in our previous studies of CVCT promotions [2, 26, 38, 39], promoters would typically invite individuals and couples within their social network.

Our client-level findings highlight the need for couple-focused HIV prevention services. We found a very high HIV prevalence (41.8 %), higher than the adult HIV prevalence estimates published in the 2012 HSRC [32]. The high prevalence of couple serodiscordance (29.5 %) was very similar to that found by the Partners in Prevention HSV-2/HIV-1 Transmission study group in this region (27.4 %) [27]. It was much higher than other countries in sub-Saharan Africa. Additionally, we found that HIV prevalence and serodiscordance differed by CVCT test site. These differences are likely explained in part by age distribution, with higher HIV prevalence and discordance found in clinics attending more couples in the 25–34 age group. Though all testing sites were located in the same general area, these differences in HIV testing outcomes can occur and implementers should be aware of this possibility. The number of couples tested also differed by site; the location of Gateway clinic on the grounds of the PMMH may have contributed to seeing the largest number of couples. A client-level measure of length of relationship was not assessed in this pilot study. This would likely be a meaningful measure especially given that 67 % of couples were not cohabiting. For a study of this nature in future, length of relationship will be useful and should be collected.

For several years, many researchers, clinicians, and sub-Saharan Africa country Ministries of Health have expressed concern regarding HIV risk and age-disparate relationships [19, 40]. We found in this study that the age disparity between couples was greatest for concordant HIV positive couples. However, due to the cross-sectional nature of our study, the temporality of the relationship between age-disparity and HIV status cannot be established. Other cross-sectional studies in Kenya, Uganda, Zimbabwe, and South Africa found that women with older partners were more likely to be HIV positive or at risk for HIV [16, 22, 24, 30]. In a population-based open cohort of 2,444 young women in KwaZulu-Natal, age disparity with their most recent sexual partner did not predict HIV acquisition among women [19]. Partner serostatus was not included in that analysis.

Previous individual HIV testing was low (50 % for men, 63 % for women). Compared to HIV positive non-pregnant women, HIV positive pregnant women were not more likely to have previously been tested. Most HIV positive persons were not on ART, including HIV positive pregnant women who had previously tested for HIV. As CVCT has been shown to increase uptake of ART among HIV positive pregnant women (PMTCT Prong 3) [14], this intervention could serve as an effective entry point to care and treatment services aimed at reducing both vertical and perinatal HIV transmission.

This pilot study compliments the GHRI-funded Canadian Sub-Saharan Africa HIV/AIDS Network, which aims to build multi-disciplinary capacity for HIV prevention research and clinical trials at seven sites in five African nations. The combination of clinic-based skills development, sociological research, and community engagement significantly enhanced Network activities that are similarly designed to use collaborative approaches to support ‘practice-based’ knowledge and skills training. Notably, this project used African experts to train Africans, an efficient South-South strategy that promotes leadership skills and learning opportunities for young African investigators. Since the Network’s focus is to build capacity by linking clinical, biomedical, and sociological researchers in Africa, it has not initiated efforts to engage HIV counselors and community volunteers or to assess intervention strategies; however, it is well situated to disseminate ‘proven’ methods to other sites in South Africa and Lesotho, who struggle with similarly devastating HIV epidemics.

## Conclusion

Implementation of standard CVCT services was found to be feasible and acceptable in Durban clinics, where burden of HIV and couple serodiscordance was found to be extremely high, using a South-South strategy. In a representative subset of the couples tested, 70 % did not know discordance was possible prior to pre-test counseling [25], and 29 % proved to be discordant couples. Long-term follow-up of couples would help to assess the effectiveness of this strategy over a period of time. The cohort of highly skilled South African counselors, researchers, and community workers generated by this project will provide an excellent local resource for continued CVCT efforts in the region.

## Abbreviations

(CVCT): Couples’ voluntary HIV counseling and testing
(ART): Antiretroviral treatment
(STI): Sexually transmitted infection
(MTCT): Mother-to-child transmission
(RZHRG): The Rwanda Zambia HIV Research Group
(HPP): IV Pathogenesis Programme
(DDMRI): Doris Duke Medical Research Institute
(SD): Standard deviation
(IPV): Intimate partner violence
(PMTCT): Prevention of mother-to-child transmission
